# Supplementary material for: The hexosamine pathway and coat complex II promote malignant adaptation to nutrient scarcity
Source: Life Sci Alliance. 2022 Apr 8;5(7):e202101334. doi: 10.26508/lsa.202101334 (PMC9008580; doi:10.26508/lsa.202101334)

Raw images of immunoblots:

1C

LUSC LUAD

GFPT1

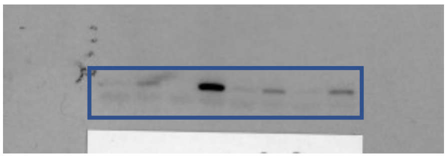

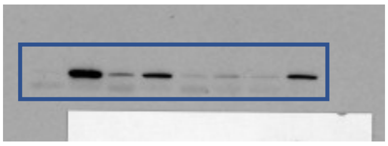

Actin

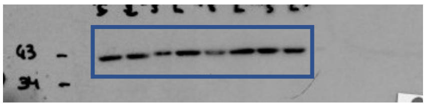

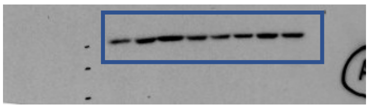

2B

GFAT1

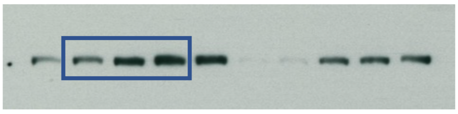

Actin

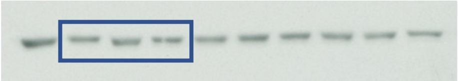

3A

RL2

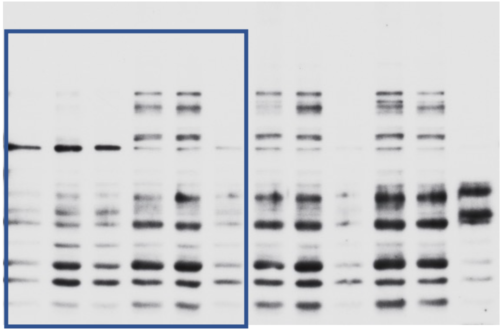

CTD110.6

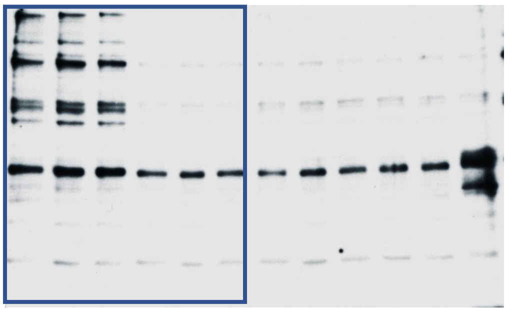

Tubulin

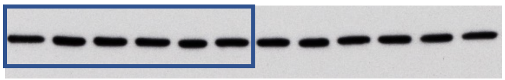

3C

CTD110.6

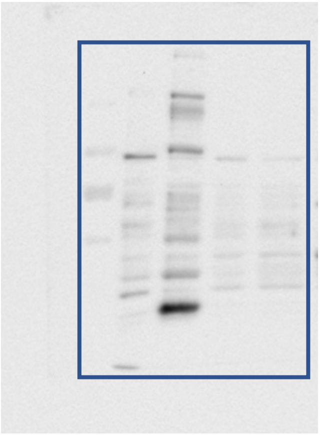

Tubulin

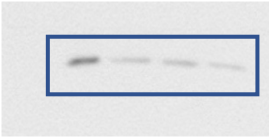

4B

CTD110.6 RL2

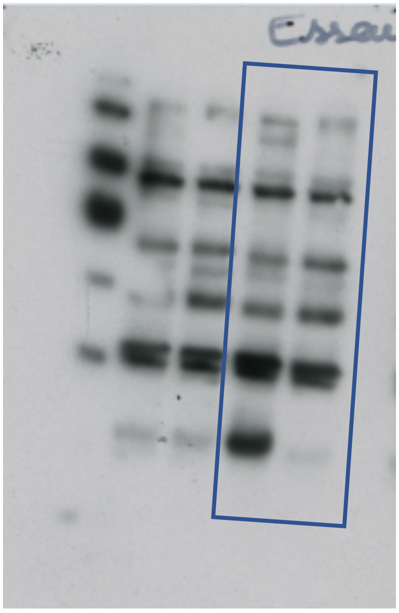

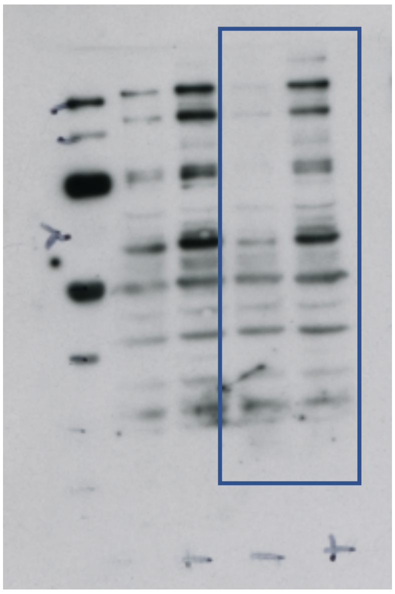

Tubulin

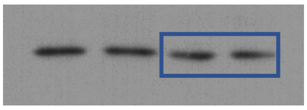

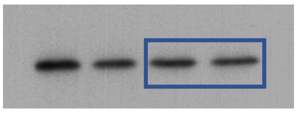

4C

Luciferase

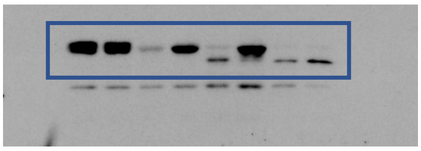

Tubulin

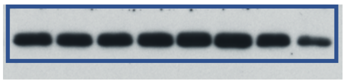

4D

Luciferase

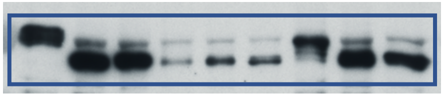

Tubulin

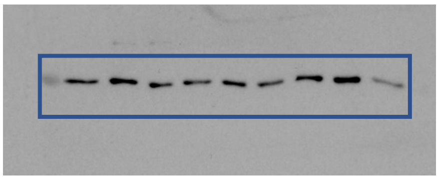

5C

EGFR

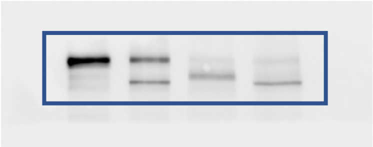

Tubulin

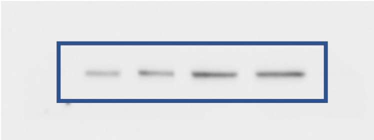

6A

EGFR

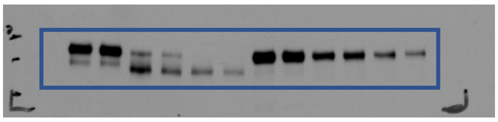

pEGFR

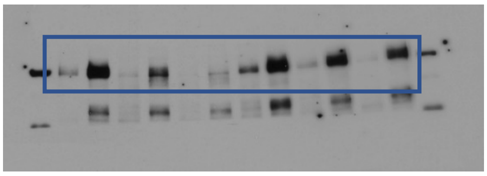

pGab1

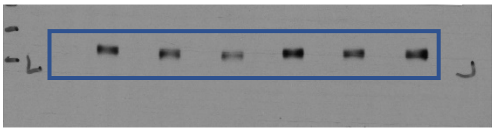

pShc

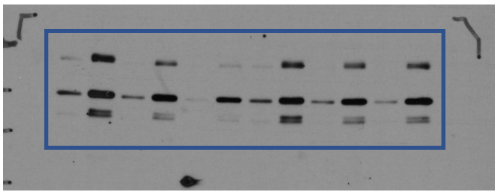

pPlcg

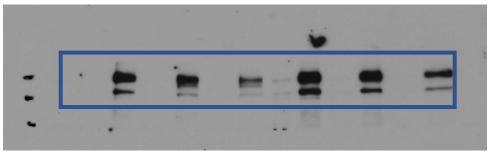

Tubulin

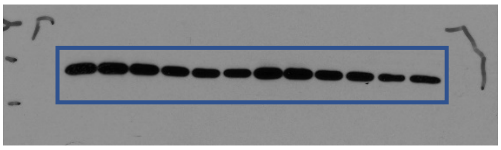

6D

EGFR

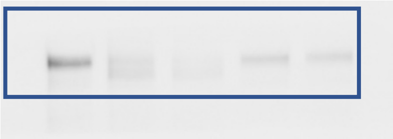

pEGFR

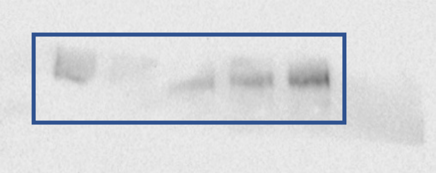

pSHC

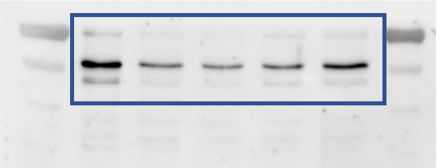

Tubulin

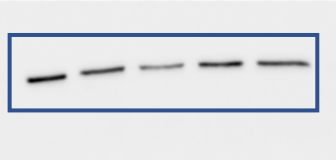

8B

Sec24D

LUSC LUAD

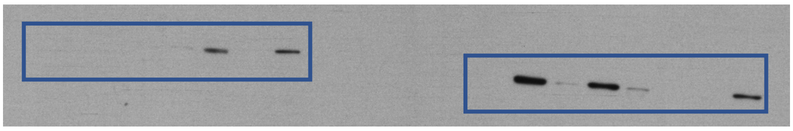

8D

Sec24D

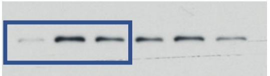

Tubulin

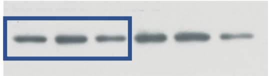

8E

Sec24D

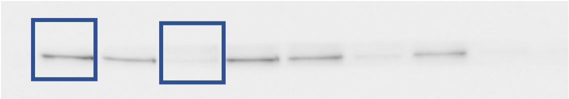

Tubulin

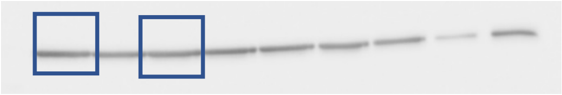

S2

LUSC LUAD

RL2

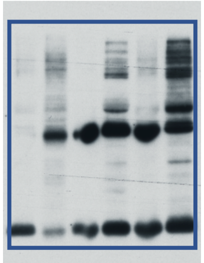

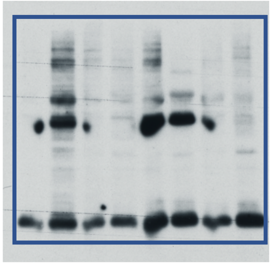

OGT

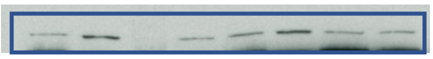

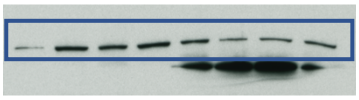

CTD110.6

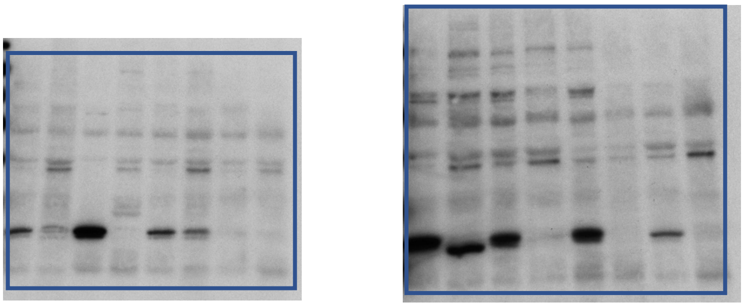

Actin

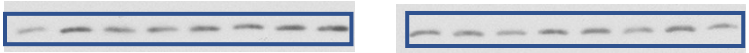

S3

EGFR

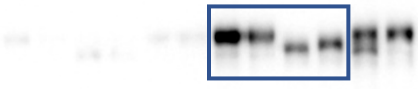

Actin

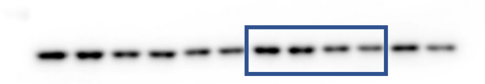

S4A

EGFR

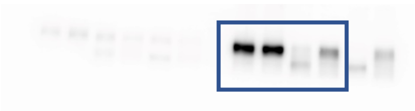

PDL1

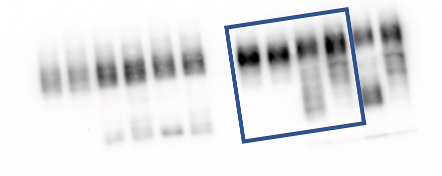

Actin

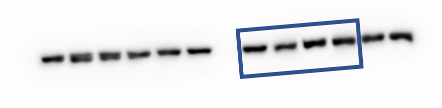

S4B

EGFR

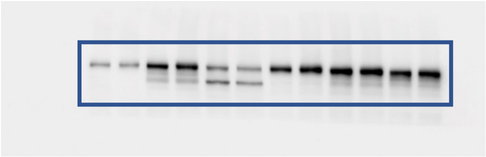

pEGFR

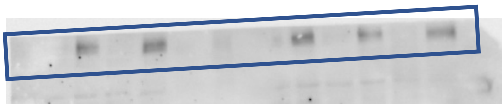

pGab1

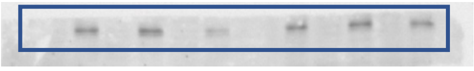

pSHC

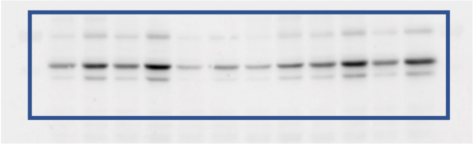

Tubulin

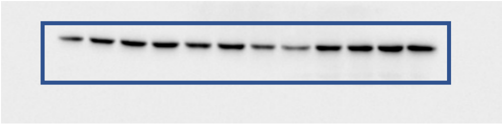

S4C – GM53

EGFR

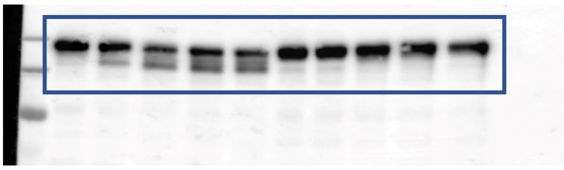

pEGFR

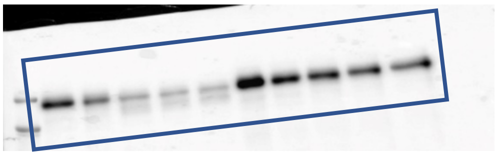

pGab1

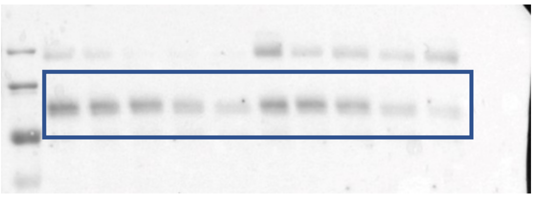

pErk1/2

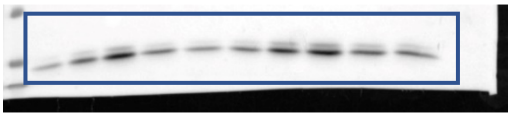

Tubulin

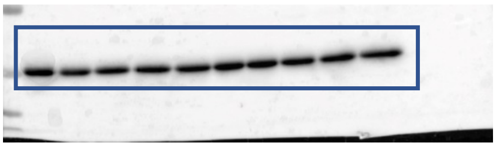

S4C-PC9

EGFR

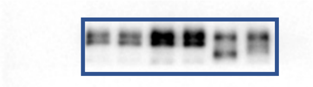

pEGFR

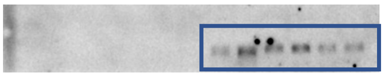

pGab1

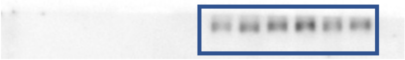

pErk1/2

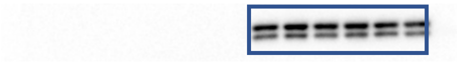

Actin

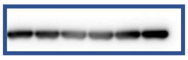

S5C

Sec24D

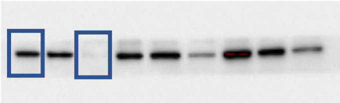

Tubulin

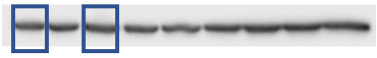

S6

NAGK

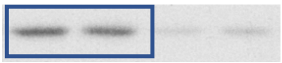

Tubulin

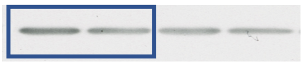

Supplement: Supplementary file 1 [file LSA-2021-01334_SdataF1_F2_F3_F4_F5_F6_F8_FS2_FS3_FS4_FS5_FS6.pdf]
